# Lipid metabolism in critically ill patients: a microdialysis study

**DOI:** 10.1186/cc9705

**Published:** 2011-03-11

**Authors:** M Theodorakopoulou, N Nikitas, S Orfanos, I Maratou, E Boutati, A Diamantakis, A Armaganidis, I Dimopoulou

**Affiliations:** 1 Attikon University Hospital, Athens, Greece; 2 Alexandra General Hospital, Athens, Greece

## Introduction

Microdialysis (MD) is a bedside *in vivo *sampling technique that permits continuous analysis of a patient's interstitial fluid chemistry without consuming blood. As the interstitial fluid bathes the cells, its composition reflects the local metabolic activities of those cells, thus reflecting intracellular metabolic changes and disorders. *In vivo *MD is performed by implanting a commercially available catheter that mimics a blood capillary at the site of interest. In this study, we used MD to assess the metabolic changes of lipids in mechanically ventilated patients with sepsis.

## Methods

Thirty-seven (21 men) mechanically ventilated septic patients were studied. All patients met the ACCP/SCCM consensus criteria for sepsis. Upon sepsis onset, an MD catheter was inserted into the subcutaneous tissue of the upper thigh. The dialysate samples were collected and analyzed immediately for glycerol using a mobile analyzer. Measurements were performed six times/day during the first 6 days from the sepsis onset. The daily mean values of the MD measurements were calculated. Blood samples were taken on the same days and were analyzed for total cholesterol, high-density lipoprotein (HDL), low-density lipoprotein (LDL), triglycerides, glycerol and free fatty acids (FFA). Results are expressed as mean ± SD. APACHE II and SOFA scores were also calculated.

## Results

Thirty-seven (21 men) critically ill septic patients with a mean (± SD) age of 65 ± 18 years were studied. APACHE and SOFA at study entry were 22 ± 4 and 8 ± 3, respectively. Sepsis was related to SIRS (*n *= 1), severe sepsis (*n *= 7) and septic shock (*n *= 29). Mortality was 43%. Serum cholesterol (81 ± 42 mg/l) along with HDL (16 ± 17 mg/dl) and LDL (63 ± 37 mg/dl) were low. Serum triglycerides (158 ± 91 mg/dl) were elevated and FFAs (0.41 ± 0.27 mmol/l) were within normal limits. Serum glycerol was high (26 ± 20 mmol/l). Interstitial glycerol was also elevated (331 ± 190 μmol/l). Serum FFAs correlated with both serum (*r *= 0.43, *P *= 0.009) and interstitial (*r *= 0.33, *P *= 0.04) glycerol.

## Conclusions

Critical care sepsis is characterized by an increase in serum and tissue glycerol and preserved FFA levels; these indicate enhanced lipolysis and an increased FFA uptake by peripheral tissues. Serum or interstitial glycerol are better indices of lipid mobilization than serum FFA levels in mechanically ventilated septic patients.

